# MiR-27 as a Prognostic Marker for Breast Cancer Progression and Patient Survival

**DOI:** 10.1371/journal.pone.0051702

**Published:** 2012-12-11

**Authors:** Wei Tang, Jiujun Zhu, Shicheng Su, Wei Wu, Qiang Liu, Fengxi Su, Fengyan Yu

**Affiliations:** 1 Department of Breast Surgery, Sun Yat-Sen Memorial Hospital, Sun-Yat-Sen University, Guangzhou, People’s Republic of China; 2 Department of Breast Surgery, The First Affiliated Hospital of Guangzhou Medical College, Guangzhou, People’s Republic of China; University of Toronto, Canada

## Abstract

**Background:**

MicroRNA-27a (miR-27a) is thought to be an onco-microRNA that promotes tumor growth and metastasis by downregulating ZBTB10. The potential predictive value of miR-27a was studied in breast cancer patients.

**Methods:**

The expression of miR-27a and ZBTB10 was examined in 102 breast cancer cases using in situ hybridization (ISH) and immunohistochemistry techniques and were evaluated semi-quantitatively by examining the staining index. The Correlation of miR-27a and ZBTB10 expression was analyed by Spearman Rank Correlation. The association of miR-27a and ZBTB10 expression with clinicopathological characteristics was analyzed using the χ^2^ test, and their effects on patient survival were analyzed by a log-rank test and the Kaplan-Meier method. Univariate and multivariate Cox regression analyses were used to evaluate the prognostic values of miR-27a and ZBTB10.

**Results:**

miR-27a was markedly up-regulated in invasive breast cancers that expressed low levels of ZBTB10 (P<0.001). A reverse correlation between miR-27a and ZBTB10 was also observed in breast cancer tissue samples (r_s_ = −0.478, P<0.001). Furthermore, the expression of miR-27a and ZBTB10 was significantly correlated with clinicopathological parameters, including tumor size, lymph node metastasis and distant metastasis (P<0.05), but not with receptor status. Patients with high miR-27a or low ZBTB10 expression tended to have significantly shorter disease-free survival times (57 months and 53 months, respectively, P <0.001) and overall survival times (58 months and 55 months, respectively, P <0.001). Univariate and multivariate analysis showed that both miR-27a and ZBTB10 were independent prognostic factors of disease-free survival in breast cancer patients (P <0.001), while only miR-27a was an independent predictor of overall survival (P <0.001).

**Conclusions:**

High miR-27a expression is associated with poor overall survival in patients with breast cancer, which suggests that miR-27a could be a valuable marker of breast cancer progression.

## Introduction

Recently, a rapidly growing number of treatment modalities have become available for the treatment of patients with breast cancer, which remarkably improve patient survival. However, tumor invasion and metastasis contribute to the great majority of breast cancer deaths. Our efforts towards the diminution of the disease should include developing novel biomarkers to use in screening for patients with a high risk of metastasis.

MicroRNAs (miRNAs) are a group of small, noncoding RNAs that regulate several biological functions. Increasing evidence supports a pivotal role for miRNAs in the multiple processes of carcinogenesis, including cell growth, apoptosis, differentiation, invasion and angiogenesis of tumor blood vessels [1], [2]. Some endothelial-specific miRNAs have been implicated in the regulation of various aspects of angiogenesis, including the proliferation, migration and morphogenesis of endothelial cells, all of which are related to cancer cell metastasis [3]. Dysregulation of miRNA expression has been found in various types of human cancers, including cancers occurring in the breast, colon, and lung, chronic lymphocytic leukemia and malignant glioma [4]–[8]. These alterations in expression are believed to be involved in cancer progression and can be prognostically indicative for human cancers [9].

MiR-27a is located at chromosome 19 and has been shown to be expressed in breast cancer, gastric adenocarcinoma and cervical cancer [10]–[12]. It has been identified as an oncogenic miRNA, and its important role in cancer development has been demonstrated in a few studies. MiR-27a had been reported to regulate cell growth and division in a dose-dependent manner [10], [13], and it might mediate the drug resistance of esophageal cancer cells [14] and ovarian cancer cells [15]. MiR-27 also promoted metastasis of human gastric cancer cell by inducing epithelial-to-mesenchymal transition (EMT) [16]. In addition, it was found to be associated with the risk of relapse in childhood ALL [17]. In breast cancer, miR-27a was involved in the apoptotic response, cell cycle checkpoints, and cellular metabolism [11], [18], [19]. Several studies observed that miR-27a exhibited oncogenic activity by directly suppressing ZBTB10/RINZF expression [11], [20], which, in turn, resulted in over-expression of transcription factor specificity protein (Sp) and Sp-dependent genes which were important for cell survival and angiogenesis [21]–[23]. ZBTB10, which was an important target of miR-27a, suppressed the expression of vascular endothelial growth factor (VEGF), VEGF receptor 1 (VEGFR1), VEGFR2 and survivin which were responsible for angiogenesis and metastasis of cancer [24], [25].Suppression of miR-27a and induced expression of the miR-27a-regulated gene ZBTB10 mediated inhibition of tumor growth in breast cancer [18] in vitro and in vivo.

These studies have demonstrated the important role for miRA-27a and its target gene ZBTB10 in regulating tumor growth, metastasis and chemotherapy resistance, which suggests that miR-27a might be a clinically useful marker for selecting high-risk cancer patients with distant metastasis.

## Methods

### Ethics

The use of tissues for this study has been approved by the Ethics Committee of Sun Yat-Sen Memorial Hospital, Sun-Yat-Sen University. At the time of initial diagnosis, all patients had provided consent in the sense that their tumor samples could be used for investigational purposes. Written informed consents were received from all participants involved in the study.

### Patients and Tissue Specimens

Paraffin-embedded tumor tissues were obtained from 124 breast cancer patients that were diagnosed and treated at the Sun Yat-sen Memorial Hospital, China, during the period from January 2001 to June 2010 and represented up to 10 years of clinical follow-up information. Of the 124 cases collected, the survival information for 102 cases (median age 50, range 23–84) was available. The tissues were acquired from the archival collections of the Department of Pathology, and used for subsequent in situ hybridization and immunohistochemistry. The clinicopathological data are illustrated in Table 1. None of the patients received any chemotherapy or irradiation prior to surgery. Histological diagnosis and scoring of all the cases were performed by 2 independent pathologists according to the WHO Histological Classification. Tumors were staged according to the TNM staging system. The disease-free survival rate of the patients was calculated from the date of resection to the date of local tumor recurrence in the form of either local or distant metastasis, while the actual survival rate was calculated to the date of death.

**Table 1:** Clinicopathological Characteristics of the Patients and the Expression of miR-27a and ZBTB10 in breast cancer.

Characteristics	cases	miR-27a expression level				ZBTB10 expression level			
		No. of lowExpression	No. of highexpression		P	No. of lowexpression		No. of highexpression	P
Age (years)					0.431				0.893
≤35	10	6(60.0)	4(40.0)			4(40.0)		6(60.0)	
35–55	61	40(65.6)	21(34.4)			20(32.8)		41(67.2)	
>55	31	16(51.6)	15(48.1)			11(35.5)		20(64.5)	
Menopause					0.967				0.654
Pre-menopausal	38	23(60.5)		15(39.5)		12(31.6)	26(68.4)		
Post-menopausal	64	39(60.9)		25(39.1)		23(35.9)	41(64.1)		
Histological grade					0.503				0.893
I	27	17(63.0)		10(37.0)		10(37.0)	17(63.0)		
II	41	27(65.9)		14(34.1)		13(31.7)	28(68.3)		
III	34	18(52.9)		16(47.1)		12(35.3)	22(64.7)		
Tumor size (cm)					0.034				0.020
≤2	52	38(73.1)		14(26.9)		12(23.1)	40(76.9)		
2–5	37	18(48.6)		19(51.4)		15(40.5)	22(59.5)		
>5	13	6(46.2)		7(53.8)		8(61.5)	5(38.5)		
N-stage					0.000				0.008
0	46	36(78.3)		10(21.7)		12(26.1)	34(73.9)		
1–3	36	18(50.0)		18(50.0)		13(36.1)	23(63.9)		
4–9	12	8(60.7)		4(33.3)		3(25.0)	9(75.0)		
>10	8	0(0.0)		8(100.0)		7(87.5)	1(12.5)		
M-stage					0.000				0.000
0	84	60(71.4)		24(28.6)		21(25.0)	63(75.0)		
1	18	2(11.1)		16(88.9)		14(77.8)	4(22.2)		
ER status					0.072				0.993
positive	32	15(46.9)		17(53.1)		11(34.4)	21(65.6)		
negative	70	46(65.7)		24(34.3)		24(34.3)	46(65.7)		
PR status					0.527				0.641
positive	17	9(52.9)		8(47.1)		5(29.4)	12(70.6)		
negative	85	52(61.2)		33(38.8)		30(35.3)	55(64.7)		
Her-2 status					0.411				0.997
positive	67	42(62.7)		25(37.3)		23(34.3)	44(65.7)		
negative	35	19(54.3)		16(45.7)		12(34.3)	23(65.7)		

ER, estrogen receptor; PR, progesterone receptor; Her-2, human epidermal growth factor receptor.

### In Situ Hybridization (ISH)

This assay was performed according to the manufacturer’s protocol (Exiqon, Vedbaek, Denmark). Briefly, thin sections (4 um thick) of paraffin-embedded specimens were deparaffinized with xylene and rehydrated with graded ethanol dilution. Sections were treated with 0.05% trypsin at room temperature for 15 minutes and re-fixed in 4% paraformaldehyde for 10 minutes. The slides were prehybridized in a hybridization solution at 51°C for 2 hours. Subsequently, 20 nmol/L of a locked nucleic acid-modified, 5′digoxigenin (DIG)-labeled oligonucleotide probe complementary to miR-27a or a scrambled control probe was added to 100 µl of the hybridization solution and hybridized at a temperature of 51°C overnight. The sections were rinsed twice in 2×standard saline citrate, followed by three washes of 20 minutes at 50°C in 50% formamide/2×standard saline citrate. Then, the samples were washed five times in PBS/0.1% Tween-20 and blocked in blocking solution (2% sheep serum, 2 mg/ml bovine serum albumin in phosphate buffered saline with Tween-20) at room temperature for 1 hour. An anti-DIG antibody (1∶1000; Abcam, Cambridge, MA, USA) was applied, and the sections were incubated at 4°C overnight. After washing in staining solution, the sections were incubated with the NBT/BCIP developing solution for 2 hours at 37°C and counterstained with nuclear fast red.

### Immunohistochemistry (IHC)

IHC was performed using standard techniques. Briefly, 4-um paraffin-embedded specimens were dewaxed in xylene and rehydrated in graded alcohols. Endogenous peroxidase was blocked using 3% hydrogen peroxide. Antigen retrieval was accomplished in citrate buffer (pH 6.0) using a microwave. Polyclonal rabbit anti-human ZBTB10 antibody (1∶50, Santa Cruz, CA, USA) was added and the samples were incubated at 4°C overnight. The sections were then treated with a secondary antibody, followed by further incubation with HSS-HRP, DAB chromogen staining and counterstaining with hematoxylin. Negative controls were obtained by replacing the primary antibody by an isotope IgG.

### Scoring of ISH and IHC

The expression of miR-27a and ZBTB10 in 102 paraffin-embedded breast invasive cancer specimens was examined and scored separately by two independent investigators blinded to the histopathological features and patient data for the samples. In each section, 5×1000 tumor cells were counted randomly, and the scores were determined by combining the proportion of positively stained tumor cells and the intensity of staining. The proportion of positively stained tumor cells was graded as follows: 0, no positive tumor cells; 1, <10% positive tumor cells; 2, 10% to 50% positive tumor cells; and 3, >50% positive tumor cells. The cells at each intensity of staining were recorded on a scale of 0 (no staining), 1 (weak staining, light blue or yellow), 2 (moderate staining, blue or yellow), and 3 (strong staining, dark blue or yellow). For tumors that showed heterogeneous staining, the predominant pattern was taken into account for scoring. The staining index (SI) was calculated as follows: staining index = proportion of positively stained tumor cells × staining intensity. Using this method, the expression of miR-27a or ZBTB10 was evaluated by the SI, scored as 0, 1, 2, 3, 4, 6, or 9. In cases of disagreement (score discrepancy >1), the slides were reexamined and a consensus was reached by the observers.

Cutoff values to define the high- and low-expression of miR-27a or ZBTB10 were chosen by a measurement of heterogeneity with the log-rank test statistic with respect to overall survival. Because the optimal cutoff SIs were identified from the current study as 6, an SI score ≥6 was taken to define tumors as high expression, and SI <6 defined tumors as low expression of miR-27a and ZBTB10.

### Statistical Analysis

All statistical analyses were performed using the SPSS 16.0 statistical software package (SPSS Inc., Chicago, IL, USA). The χ^2^ test was used to analyze the relationship between miR-27a or ZBTB10 expression and the clinicopathological characteristics. Bivariate correlations between study variables were calculated via Spearman’s rank correlation coefficients. Survival curves were plotted by the Kaplan-Meier method and compared by the log-rank test. The survival data were evaluated by univariate and multivariate Cox regression analyses. In all cases, a P<0.05 was considered statistically significant.

## Results

### Expression Levels of miR-27a and ZBTB10 in Invasive Breast Cancer

For the specific identification of miR-27a in tissue sections using ISH, we employed high-affinity LNA-containing DNA oligomers labeled at the 5′-end with DIG. Immunohistochemistry was performed to evaluate the expression level of ZBTB10 using polyclonal rabbit anti-human ZBTB10 antibody. The clinicopathological parameters of the 102 breast cancer patients used in this study are described in Table 1. Of these patients, 18 cases (17.6%) had distant metastasis.

**Figure 1:**
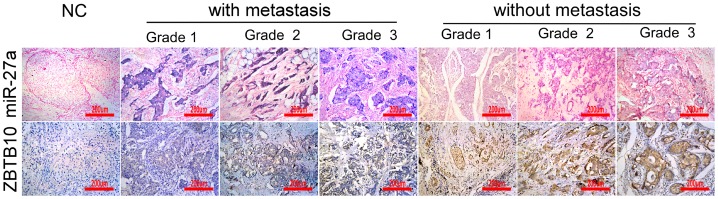
In-situ hybridization for miR-27a and immunohistochemical staining for ZBTB10 in metastasic versus non-metastasic breast cancer specimens (×200).

We found that miR-27a was higher expressed in breast invasive cancers with distant metastasis, compared with non-metastatic cancers, in spite of histological grade. However, the level of ZBTB10 expression was lower in breast cancers patients with distant metastasis,compared with non-metastatic cancers (Figure 1). MiR-27a, as well as ZBTB10, was expressed in the cytoplasm of most neoplastic tumor cells. The expression difference between metastatic and non-metastatic invasive breast cancers determined by the SI was statistically significant (P<0.001; Table 1).

In addition, Spearman order correlation analysis showed that ZBTB10 expression in invasive breast cancer was inversely correlated with the miR-27a level (r_s_ = −0.478, P<0.001).

**Figure 2:**
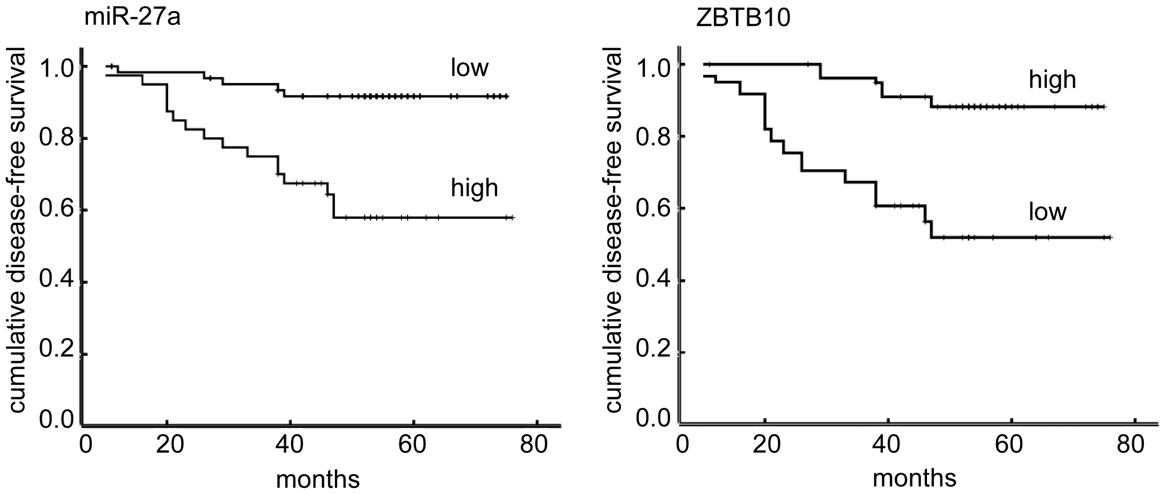
Kaplan–Meier curves showing the relationship between miR-27a and ZBTB10 expression and disease-free survival in patients with breast cancer. Patients expressing high levels of miR-27a (A) or low levels of ZBTB10 (B) have a significantly shorter survival (P<0.0001).

**Figure 3:**
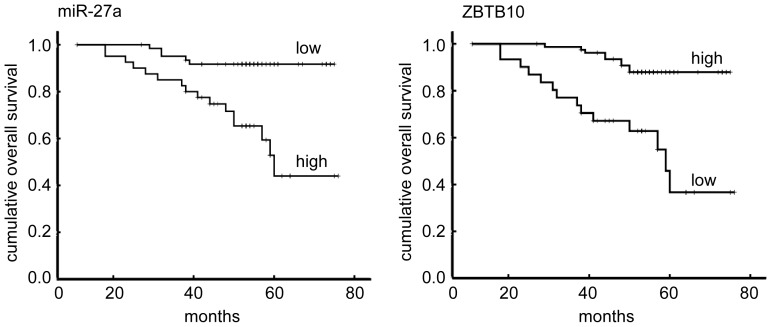
Kaplan-Meier overall survival curves of breast cancer patients in association with miRNA-27a expression levels (A) and ZBTB10 expression levels (B). The difference between the curves was significant (P<0.0001).

In contrast, higher ZBTB10 expression was associated with better disease-free survival (HR:1.395, 71 months, 95% confidence interval, 67.87 to 73.34, P <0.001) and overall survival (HR:1.205, 71 months, 95% confidence interval, 68.85 to 73.58, P<0.001). Disease-free survival (HR: 3.309, 53 months, 95% confidence interval, 46.32 to 59.29) and overall survival (HR: 2.83, 55 months, 95% confidence interval, 49.41 to 60.50) were reduced in the low ZBTB10 expression subgroup.

**Table 2:** Univariate and Multivariate Analyses of Different Prognostic Parameters on Breast Cancer Disease-free Survival Rates.

	Univariate analyses		Multivariate analyses		
	P	Regression coefficient (SE)	P	Relative risk	95% Confidence interval
Age	0.893	−0.05 (0.371)			
Menopause	0.915	0.048(0.449)			
Histological grade	0.745	0.095 (0.291)			
T-stage	0.000	1.151(0.292)	0.001	3.197	1.653–6.185
N-stage	0.016	0.497(0.207)			
ER status	0.935	−0.038(0.463)			
PR status	0.333	0.72(0.744)	0.054	4.778	0.973–23.478
Her-2 status	0.055	0.84(0.437)	0.012	3.373	1.300–8.750
miR-27a	0.001	1.728(0.513)	0.025	3.573	1.176–10.860
ZBTB10	0.000	−1.846(0.485)	0.019	0.268	0.089–0.802

(SE) standard error; multivariate analysis; Cox proportional hazard regression model, stepwise forward LR.

**Table 3:** Univariate and Multivariate Analyses of Overall Survival Rates in Patients with Breast Cancers by Cox-Regression Analysis.

	Univariate analyses		Multivariate analyses		
	P	Regression coefficient (SE)	P	Relative risk	95% Confidence interval
Age	0.851	−0.068 (0.361)			
Menopause	0.872	0.072(0.45)			
Histological grade	0.721	0.104(0.292)			
T-stage	0.000	1.2(0.293)	0.000	3.013	1.645–5.519
N-stage	0.016	0.494(0.204)			
ER status	0.958	−0.024(0.463)			
PR status	0.358	0.684(0.744)			
Her-2 status	0.028	0.977(0.443)			
miR-27a	0.001	1.739(0.513)	0.003	4.575	1.665–12.569
ZBTB10	0.000	−1.774(0.484)			

(SE) standard error; multivariate analysis; Cox proportional hazard regression model, stepwise forward LR.

### Correlation of miR-27a and ZBTB10 Expression with Clinicopathological Characteristics of Breast Cancer

To further evaluate whether miR-27a high-expression was linked to the clinical progression of breast cancer, we analyzed the association of miR-27a and ZBTB10 expression with the clinicopathological status of breast cancer patients (Table 1). The miR-27a level was closely associated with tumor size, lymph node metastasis and distant metastasis of the patients. Tumors of larger size or metastasis expressed higher levels of miR-27a, suggesting that miR-27a up-regulation was associated with tumor progression. However, no significant correlation was observed between miR-27a expression and age, menopause, histological grade or hormone receptor status. On the contrary, ZBTB10 expression was negatively correlated with tumor size, lymph node metastasis and the distant metastasis of breast cancers. However, the expression level of ZBTB10 was not significantly associated with age, menopause, histological grade or hormone receptor status.

### MiR-27a and ZBTB10 Expression is Associated with Poor Survival of Invasive Breast Cancer Patients

A log-rank test and Kaplan-Meier analysis were used to calculate the effect of miR-27a and ZBTB10 expression on patient survival (Figure 2, 3). Specifically, the median disease-free survival and overall survival time of patients whose tumors expressed high levels of miR-27a was only 57 (HR:2.703, 95% confidence interval, 51.51 to 62.10) and 58 months (HR:2.389, 95% confidence interval, 53.63 to 63.00), respectively, whereas the median survival time of those with low levels of miR-27a expression was 71 (HR:1.677, 95% confidence interval, 67.88 to 74.46, P<0.001) and 72 months (HR:1.474, 95% confidence interval, 68.68 to 74.46, P<0.001), respectively.

### Univariate and Multivariate Analyses of Prognostic Variables in Breast Cancer Patients

Univariate and multivariate analyses were performed to determine the prognostic value of clinicopathological variables. The univariate analyses indicated that miR-27a expression, as well as T-stage, N-stage and ZBTB10 expression, was significantly associated with disease-free survival (P = 0.001) of breast cancer patients (Table 2). Furthermore, strong miR-27a and weak ZBTB10 expression were correlated with poorer disease-free survival in multivariate analyses (P = 0.025).

As shown in Table 3, T-stage (P < 0.001), N-stage (P = 0.016), Her-2 status (P = 0.028), miR-27a expression (P = 0.001) and ZBTB10 expression (P < 0.001) were all significant prognostic indicators of overall survival in univariate analyses. However, in the multivariate analyses, only miR-27a expression (P = 0.003) and T-stage (P < 0.001) were independent prognostic factors, while none of the other clinicopathological variables had an independent prognostic impact.

## Discussion

An increasing number of in vitro studies have demonstrated an important role for miR-27a in regulating tumor growth, metastasis and chemotherapy resistance. However, little is known about the relationship between the expressions of miR-27a in human breast cancer with the prognosis of breast cancer patients. In the present study, we found that breast invasive cancers with higher miR-27a expression tended to have distant metastasis and over-expression of miR-27a was associated with shorter disease-free survival and overall survival of breast cancer patients. Both of the univariate analyses and multivariate analyses indicated that miR-27a expression was an independent prognostic factor for breast cancer progression.

Several recent studies have demonstrated that the expression of miR-27a is up-regulated in several types of solid tumors, including colon, gastric, cervical and breast cancers [10], [12], [24], [26]. The widespread overexpression of miR-27a in cancer has led to the belief that miR-27a is an oncogenic microRNA. Cell culture and animal experiments support this speculation, showing that the down-regulation of miR-27a expression can suppress cell proliferation and slow tumor growth. In gastric cancer cells, the reduction of miR-27a inhibited cell growth in both in vitro and nude mice assays [27]. MiR-27a might mediate cell proliferation by the regulation of cyclin D1 and p21. In addition, it could promote the migration of pancreatic cancer cells by targeting Sprouty2 [28] and increase endothelial cell sprouting by regulating the expression of the angiogenesis inhibitor semaphorin 6A (SEMA6A) [29]. In addition, miR-27a plays an important role in mediating drug resistance by targeting multiple drug-resistance related genes. MiR-27a modulated MDR1/P-glycoprotein expression in human ovarian cancer cells by targeting HIPK2 [15] and could reverse the multidrug resistance of esophageal squamous cell carcinoma through regulation of MDR1 and apoptosis [14].

This study focused on the potential relationship between the expression level of miR-27a and various clinicopathological characteristics of breast cancer patients, as well as disease-free survival and overall survival. It is worth noting that high levels of miR-27a appear to be significantly correlated with tumor size, lymph node metastases, distant metastasis and poor prognosis in patients with breast cancer. MiR-27a was up-regulated in patients presenting with metastases, suggesting that its up-regulation was acquired in the course of tumor progression and, in particular, during the acquisition of metastatic potential. Our results showed that miR-27a and ZBTB10 expression were not correlated with receptor status. On the contrary, it was reported that miR-27a indirectly regulates estrogen receptorα expression and hormone responsiveness in MCF-7 breast cancer cells through the suppression of ZBTB10 [30]. To understand these conflicting results, the difference between clinical observation and in vitro experiments should be considered. After dividing the patients by the cut-off method, a multivariate Cox proportional hazard regression analysis revealed that miR-27a overexpression had a significantly worse prognostic impact (P = 0.003) on the overall survival of breast cancer patients independent of tumor size (P = 0.000). These results indicate that, as an independent risk factor, miR-27a could serve as a prognostic marker for the survival of patients. To date, several studies have revealed the prognostic significance of miR-27a overexpression in various carcinomas, such as gastric cancer [31], acute lymphoblastic leukemia [17] and osteosarcoma [32]. To the best of our knowledge, our research may be the first report to evaluate the prognostic value of miR-27a in breast cancer.

Several tumor suppressor genes have been identified as targets of miR-27a regulation, including ZBTB10 [24], [33], FOXO1 [34] and prohibitin [10]. By downregulating ZBTB10, miR-27a could increase the expression of the specificity protein (Sp) transcription factors Sp1, Sp3 and Sp4 and several Sp-regulated genes/proteins, including vascular endothelial growth factor, survivin, cyclin D1 and fibroblast growth factor receptor-3. All of these genes encode tumor suppressors that are involved in breast cancer migration and invasion. Correspondingly, miR-27a also plays a role in invasion and metastasis [33], [35], [36].

Our results showed that expression of miR-27a was lower and the expression of ZBTB10 was higher in the non-metastatic group compared to the metastatic group. Like miR-27a, the difference in the expression of ZBTB10 between metastatic and non-metastatic breast cancers was statistically significant. In addition, Spearman order correlation analysis showed that ZBTB10 expression in breast cancer was inversely correlated with the miR-27a level. ZBTB10 levels were closely associated with tumor size, lymph node metastasis and distant metastasis of the patients. This may contribute to the ZBTB10 regulation of Sp, which is related to tumor growth and metastasis. However, we did not find that ZBTB10 had prognostic importance in the multivariate Cox proportional hazard regression analysis. These results suggest that miR-27a promotes tumor growth and metastasis by targeting not only ZBTB10 but also other tumor suppressor genes and that ZBTB10 alone does not demonstrate any prognostic value.

In summary, the results of our study indicate that the expression of miR-27a is strongly correlated with the clinical stages and overall survival times of patients with breast cancer, providing evidence that up-regulation of miR-27a might play an important role in the progression of the disease. The study results are consistent with the literature and support the notion that miR-27a is an oncogenic microRNA that induces effects by regulating ZBTB10.
